# Spatial Visualization Supports Students’ Math: Mechanisms for Spatial Transfer

**DOI:** 10.3390/jintelligence11060127

**Published:** 2023-06-20

**Authors:** Tom Lowrie, Tracy Logan

**Affiliations:** STEM Education Research Centre, University of Canberra, Canberra 2617, Australia; tracy.logan@canberra.edu.au

**Keywords:** spatial reasoning, math, spatial digital training, spatial visualization

## Abstract

The present study conducted a randomized control trial to assess the efficacy of two spatial intervention programs aimed to improve Grade 4 (N = 287) students’ spatial visualization skills and math performance. The first treatment (N = 98) focused on isolated spatial training that included 40 min of digital spatial training across fourteen weeks. The second treatment (N = 92) embedded spatial visualization skill development into math lessons, along with the digital spatial training that provided practice of the newly acquired skills. A business-as-usual group acted as a control (N = 97). Engagement with the embedded intervention program (i.e., both lessons and digital training) showed large additive effects, highlighting the role of spatial reasoning tools to support the transfer of spatial reasoning to math. The isolated intervention program with the digital spatial training had a transfer effect on math, compared to a business-as-usual control, while spatial reasoning improvements for this group were mixed. The spatial skills targeted in the digital training had a mediation effect on math performance, despite not increasing in the pre–post-test design. The effects of the digital training cohort were moderated by initial spatial skill, with students with lower spatial reasoning making the least gains in math.

## 1. Introduction

Spatial skills have broad implications for mathematics understanding, achievement, and pursuit of STEM careers (Hegarty and Kozhevnikov 1999; Sorby et al. 2013; Wai et al. 2009). In their meta-analysis, Xie et al. (2020) demonstrated strong associations between spatial reasoning and mathematics and that these positive associations are not moderated by any one type of spatial reasoning or age. These findings highlight that improvement in spatial reasoning will have positive outcomes for mathematics understanding. 

Recently, training studies have focused on leveraging the development of spatial skills for mathematics outcomes. However, despite positive trends, many questions remain about the conditions most conducive to successful transfer between spatial training and mathematics achievement (Hawes et al. 2022; Lowrie et al. 2020). In this study, we addressed the critical question of the role of spatial curriculum versus skills training in this relationship by implementing spatial visualization training either in the form of digital games or a learning program combined with digital games. Furthermore, to account for teacher knowledge and confidence in spatial reasoning (Atit and Rocha 2020), all educators, including control group educators, were offered professional learning on the importance of spatial reasoning. This paper reports on the outcomes of this novel intervention design and the implications for incorporating spatial skills development into real-world classroom learning. 

### 1.1. Intervention Studies

Across much of the literature, intervention studies fall into two main categories, as defined by Hawes et al. (2023), isolated and embedded approaches. Isolated approaches refer to repeated practice of a defined skill, such as spatial scaling, whilst embedded approaches consider spatial skills and related mathematics content simultaneously. Within the isolated approach, initial studies of spatial intervention effects on mathematics have been mixed (Cheng and Mix 2014; Hawes et al. 2015). The first published experimental study of spatial training with a transfer to mathematics was by Cheng and Mix (2014). In this study, they trained mental rotation for 40 min and found improvements in 6–8-year-old children’s ability to complete missing term problems (e.g., 4 + __ = 7). They posited possible reasons for this transfer, such as an improved ability to rotate equations into more conventional formats or an increase in visuospatial working memory capacity. Hawes et al. (2015) attempted a replication and extension of this study with digital mental rotation training occurring three times a week (15–20 min per session) over six weeks. They found improvements in 2D mental rotation, but they did not find transfer to other spatial or mathematics tasks. 

Within the embedded approaches, longer-term instruction-based interventions subsequently produced a broader range of impacts on spatial and mathematics skills (Hawes et al. 2017; Lowrie et al. 2017; Mulligan et al. 2020). Hawes et al. (2017) reported on a 32-week intervention with a spatialized geometry program implemented by teachers, which included professional development on the teaching and learning of spatial reasoning. The program included spatialized geometry content (including concepts of symmetry, rotations, area measurement, and proportional reasoning) and quick challenge spatial activities designed to develop spatial visualization skills. In this study, they reported improvements in spatial language, visual-spatial geometry (e.g., paper folding and 2D shapes), symbolic comparison (i.e., numbers) as well as 2D mental rotation. Meanwhile, Lowrie et al. (2017) replaced standard geometry instruction with a 10-week spatial program developed in collaboration with teachers. The program focused on mental rotation, spatial visualization, and spatial orientation and resulted in overall improvements in mathematics, particularly geometry and measurement, relative to business-as-usual controls. In this study, the impact on number problems was negligible; however, the participants had similar gains in their number knowledge compared to the control group. Similarly, Mulligan et al. (2020) embedded spatial transformation and structuring skills into a longitudinal mathematics program with students across Grades 3 and 4. This study reported improvement for the intervention students across patterns and structures and general spatial reasoning skills, with students demonstrating advanced spatial thinking but did not find transfer to general mathematics. 

Many studies have since reported different methods of spatial training with the goal of improving mathematics with varying degrees of success and transfer (e.g., Gilligan et al. 2019; Lowrie et al. 2019, 2021; Mix et al. 2021). These studies recently culminated in a meta-analysis that reported the global benefit of spatial training on mathematics, with a Hedges’s g of .279 (Hawes et al. 2022). However, the authors of the meta-analysis were still unclear about the nature of transfer, that is, under which conditions transfer was most likely to occur. They reported two moderator effects, age and the use of physical resources, meaning that transfer effects were greater for older children and for interventions that included physical resources (regardless of whether the training involved worksheets or digital training). 

Hawes et al. (2022) did not explore the use of pedagogy in their analysis. Based on the studies included in the meta-analysis and the existing literature, the use of spatial pedagogy seems rare, with a general focus on training of spatial skills (e.g., mental rotation or spatial visualization; Gilligan et al. 2019; Mix et al. 2021) or spatial tools (e.g., gesture and language; Bower et al. 2020; Bower and Liben 2021). Successful intervention studies by Lowrie et al. (2018a, 2018b, 2019, 2021) and Mulligan et al. (2020) have attempted to overcome this limitation by including spatial pedagogy combined with professional learning (PL) around the benefits of spatial reasoning. However, it is possible that the PL delivered to the intervention program teachers played a role in their findings. Other studies have overcome this by delivering parallel PL to the control groups without the same intervention benefits (Hawes et al. 2017). 

### 1.2. The Nature of Transfer

Many theoretical accounts have been compiled to explore the nature of transfer between spatial skills development and mathematics (Resnick et al. 2020; Sinclair and Bruce 2015; Mix and Battista 2018). Yet, the exact circumstances that facilitate the transfer are still unknown; Gagnier and Fisher (2020) refer to this as the “black box of translation” (p. 1). The problem is not just about the mechanism that allows the transfer to take place but how to make the skills training, currently conducted in controlled environments, accessible for teachers and beneficial for students (Green and Newcombe 2020). 

Transfer in the context of spatial training and mathematics has been classified in degrees. Gilligan et al. (2019) reported near transfer for both mental rotation and spatial scaling training, that is, improvements in test performance on the skills trained in the intervention. However, the nature of intermediate and far transfer differed according to the training condition. For mental rotation training, improvements were found in spatial scaling (intermediate transfer) and missing term and geometry-shape problems (far transfer). For the spatial scaling group, there were no intermediate transfer effects, but there were improvements in number line estimation and geometry shape (far transfer). It is interesting to note that neither group showed improvements in symmetry problems relative to the control group. One conclusion to be reached from this is that the mechanism of transfer is impacted by the content of the training materials. Gilligan et al. (2019) delivered their intervention in the same way for both training conditions, but the outcomes varied. 

Stieff and Uttal (2015) were cautiously optimistic about the nature of transfer from spatial training to STEM outcomes. They argued that although the outlook is promising, more work is needed to understand the effectiveness of spatial training with realistic outcomes from students. Resnick and Stieff (forthcoming) hypothesized that inconsistencies in transfer to date might be due to the stringent and narrow conditions of training studies. They proposed that a more practical and sustainable solution might be to develop skills across a variety of settings and tasks, thereby providing more opportunities to incorporate learning across contexts. This model may hold some clues as to why the embedded interventions (e.g., Hawes et al. 2017; Lowrie et al. 2017, 2019, 2021) demonstrate broader transfer. When spatial skills training is embedded in real-world, contextually rich activities, the results may be more meaningful in terms of student learning and thus more impactful and transferable (Resnick and Stieff, forthcoming).

### 1.3. Spatial Visualization

In their meta-analysis, Hawes et al. (2022) reported that a large proportion of training studies focused on spatial visualization training. Spatial visualization is often dubbed a “catch-all” phrase for complex spatial tasks that do not fit naturally within a clear category (Battista et al. 2018; Linn and Petersen 1985; Patahuddin et al. 2018). However, unlike other spatial skills that may act as a precursor to higher-level mathematical content (e.g., mental rotation; Battista et al. 2018, or mental transformation; Gunderson et al. 2012), many tasks that comprise spatial visualization sit squarely within mathematics curricula in elementary grades (e.g., symmetry, 2D to 3D transformations; Hawes et al. 2017; Ramful et al. 2017). The fact that this level of mathematics draws explicitly on spatial visualization skills provides an opportunity to value-add to existing curricula by drawing on knowledge from cognitive and learning sciences (Stieff and Uttal 2015) in mathematics instruction. 

#### 1.3.1. Reflection and Symmetry

There is a pervasive notion that symmetry is about folding (Leikin et al. 2000; Ng and Sinclair 2015; Ramful et al. 2015); in fact, that is how it is measured in psychology (Ekstrom et al. 1976). However, more broadly, it involves reflections and relations to the line of symmetry itself and between components; for example, the slope of the incline line of symmetry changes the orientation of the objects being reflected (Ramful et al. 2015). Reflection and symmetry are inextricably linked and are critical for higher-level mathematics (Clements and Battista 1992; Leikin et al. 2000; Ng and Sinclair 2015).

Symmetry is not just a mathematical construct but intrinsic to mathematics (Sinclair 2004). Consider the balance between the sides of an equal sign; symmetry offers a unique perspective to consider equivalence, not just as a step in an operation (Patahuddin et al. 2018). The function of the equal sign when solving equations is a concept difficult for many school students to grasp when they remain focused on operations (Kieran 1981). Cheng and Mix (2014) even went so far as to offer spatial training as a potential reason for the improvement in missing term problems due to this effect. 

In a study of pre-service educators teaching eighth-grade students about the concept of symmetry, many pre-service educators reported initial apprehension and reluctance around the topic of symmetry (Leikin et al. 2000). However, the series of lessons in the study focused on different applications of symmetry, incorporated physical representations (in terms of folding and drawing), and linked concepts to real-world examples. At the completion of the study, the authors reported positive responses from the pre-service educators, with many expressing their new view of symmetry as something embedded in nature and the world around them, as well as mathematics, and that symmetry can be used to help solve many mathematics problems. 

#### 1.3.2. 2D to 3D Transformations

Spatial visualization includes the process of mentally moving internal parts of spatial configurations, often in complex, multi-step manipulations (Ramful et al. 2017). One example of this is the transformation of two-dimensional (2D) shape representations into three-dimensional (3D) object representations, commonly referred to in the literature as mental folding or 3D mental folding (Chen and Yang 2023; Harris et al. 2013). Within the literature, measures of this type of spatial skill include the Differential Aptitude Test (DAT), Space Relations Subset (Bennett et al. 1947) and the Surface Development Test (Ekstrom et al. 1976), where test takers are asked to visualize a 2D shape of a plain sheet that, by proper folding, could be converted into the shape of the 3D solid figure. Often, decisions about the relations between the corners and edges of the folded object are required. The 3D version of mental folding has received much less attention compared to its 2D form (Harris et al. 2013), where many studies utilize the 2D form of the paper folding test (Ekstrom et al. 1976) or a version of this, such as the Children’s Mental Folding Task (Harris et al. 2013). However, the 3D form aligns directly with mathematics geometry curricula as students are often asked to connect 3D objects with their 2D nets (Wright and Smith 2017). Within mathematics, nets are defined as “Plane figures that can be folded to form a polyhedron. More specifically, two-dimensional representations comprising joined shapes (the faces) that can be folded (along edges) to form the object” (Australian Curriculum, Assessment, and Reporting Authority (ACARA) 2022). 

This definition suggests that students are being asked to complete very similar mental folding actions as those required in the 3D mental folding task within their mathematics learning. Research with elementary-aged children on 3D mental folding tasks found they were able to identify some standard nets of cubes that had three or four squares in a row (Wright and Smith 2017), recognize corresponding edges on cubes (Ramful et al. 2017), and select opposite faces of a cube (Burte et al. 2019). However, research on the training of this type of mental folding is limited. 

### 1.4. The Present Study

In the research to date, most isolated spatial training interventions have been administered by a member of the research team, with the training presented either with individuals or small groups outside of classroom contexts. Embedded spatial training interventions have traditionally been conducted within whole-class contexts or situated within the participants’ standard classroom practices, usually by a member of the research team. Although more recent studies have included the classroom teacher in the delivery (e.g., Hawes et al. 2017; Lowrie et al. 2018b), few studies, if any, have compared isolated and embedded training under typical classroom conditions. 

To this point, the present study examined the efficacy of both isolated and embedded training approaches in the research design. The isolated intervention was undertaken with digital spatial training made available to the classroom teachers through a bespoke digital platform. The embedded intervention was designed within a pedagogical framework that ensured participants’ classroom teachers could administer the program. The Experience-Language-Pictorial-Symbolic-Application (ELPSA) learning framework (Lowrie et al. 2018a) draws on well-known sociological and psychological understandings of learning and was used as the basis for the program design (see Lowrie et al. 2018b for further explanation of the framework and Appendix A for an overview of the embedded spatial training intervention). The framework promoted learning as an active process in which individuals develop understanding through discrete, scaffolded activities using hands-on materials and social interactions. The sequence provided a logical structure to scaffold, reinforce and apply knowledge and concepts (Lowrie et al. 2018a). The embedded intervention also included the use of digital spatial training. 

Given that both isolated and embedded approaches were used in this study, the following research questions were considered:

- To what extent do the spatial training interventions (i.e., the isolated and embedded training programs) facilitate near transfer (to spatial skills) and far transfer (mathematics understandings)?
- If transfer occurs, can any transfer mechanisms be determined from the different training approaches?

## 2. Methods

### 2.1. Participants

Participants (N = 287) were drawn from 15 Grade 4 elementary school classrooms from a school jurisdiction in metropolitan Sydney. As part of the study design, the students (mean age = 9 years, 8 months) remained in their usual class throughout the intervention. Information and consent forms were sent to all families of children attending a participating school. Only students with full parental consent to have test-score results utilized are reported in this manuscript. 

All schools in the study were drawn from average sociodemographic areas. In Australia, the socioeconomic advantage of a school is measured by the Index of Community Socio-Educational Advantage (ICSEA) scale. A score (Mean = 1000, S.D = 100) is produced for each school, based on Australian Bureau of Statistics (ABS) data, school location, and the proportion of Indigenous students enrolled in the school as well as data on parents’ self-reported income, qualifications, and occupation. Thus, a value on the index corresponds to the average level of educational advantage of the school’s student population relative to those of other schools. The ICSEA scores for all schools in the study ranged from 1039 to 1103, and there were no statistically significant differences between the schools in the three groups, t(8) = .016, *p* = .98.

### 2.2. Study Design

An expression of interest was sent to schools from the educational jurisdiction to recruit the teachers. The teachers from the participating schools undertook a six-hour professional learning session with the authors that was aimed at (i) highlighting the importance of spatial visualization in the curriculum, (ii) identifying assessment tasks that require spatial visualization skills, and (iii) supporting teachers’ spatial skill development. After the completion of the professional learning day, teachers were then randomly assigned to one of three groups, namely: (a) business-as-usual control, (b) isolated digital spatial training, and (c) embedded spatial training program. 

The study ran across the final two terms of the school year (approximately 17 weeks, including professional learning and testing). Table 1 outlines the design of the study. The intent of the design was for the two intervention groups to replace small elements of their regular curriculum with either isolated spatial training (Group B) or the embedded spatial program (Group C) to determine the extent to which the intervention approach facilitated transfer to mathematics. Consequently, the overall time spent on mathematics in a week was equivalent to approximately four hours a week. Groups A and B both continued with their regular mathematics programs, with Group B being asked to replace approximately 40 min of Geometry and Measurement learning with the isolated digital training. Group C was asked to replace their Geometry and Measurement content with the embedded spatial program, which aligned with the curriculum outcomes. We acknowledge that the isolated training that occurred in Group B might have promoted deeper Geometry and Measurement learning across the intervention. To this point, these teachers might have changed their pedagogical approaches in ways that additionally supported learning. Similarly, the BAU Group A could be considered an active control given that the classroom teachers undertook professional learning that exposed them to learning activities that were spatial in nature.

#### 2.2.1. The Business-as-Usual Group (Group A)

The business-as-usual (BAU) groups’ learning activities were drawn from the Australian Curriculum guidelines (Australian Curriculum, Assessment, and Reporting Authority (ACARA) 2022). This group completed regular mathematics classes for approximately 4 h a week. The content covered by the control group teachers included concepts associated with numbers and algebra, geometry and measurement, and statistics and probability. For students in Grade 4, any opportunity for the development of students’ spatial reasoning skills would be covered in the geometry strand of the mathematics curriculum, particularly content associated with ‘shape’ and ‘location and transformation’. For example, students compose and decompose 2D shapes, create and interpret maps, use the direction to interpret maps and create symmetrical patterns. 

#### 2.2.2. The Isolated Intervention Group (Group B)

**Figure 1:**
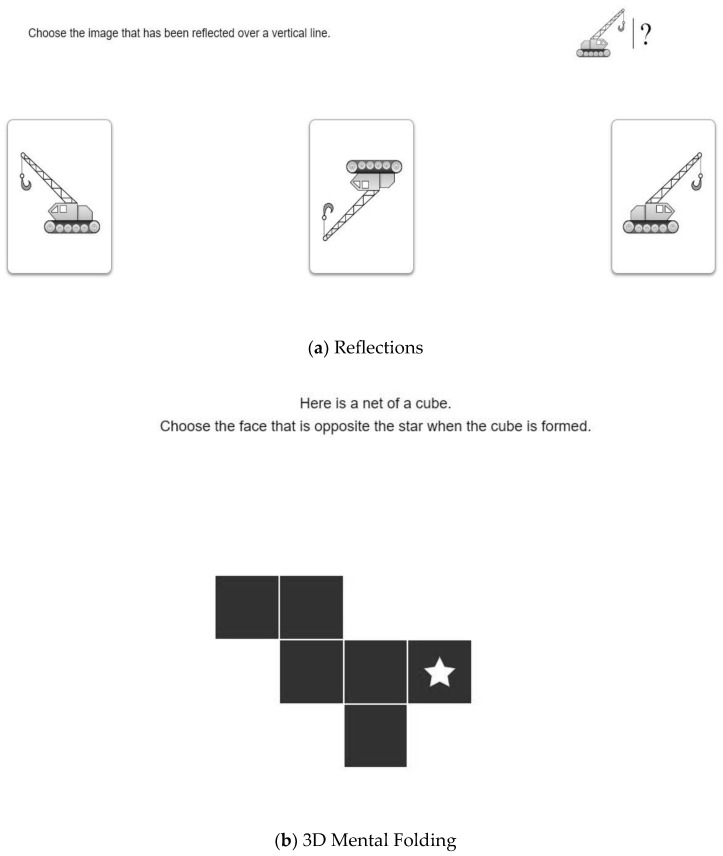
Examples of the digital training activities for (**a**) reflections and (**b**) 3D mental folding.

The Group B cohort engaged with the bespoke digital spatial training for approximately 40 min per week over the course of the 14 weeks. The isolated training group continued with their regular mathematics instruction along with the digital spatial training. These digital training activities were implemented by classroom teachers at a time of their choosing across the week. The digital training encouraged participants to practise spatial skills aligned with reflections and 3D mental folding in a digital environment. Participants engaged in seven weeks of reflections and seven weeks of 3D mental folding. As identified in the literature section, the focus on reflections considered the spatial movements of reflections in relation to the line of symmetry itself and between components, as opposed to the general mental act of folding. Few intervention studies have examined training 3D mental folding skills, and given its explicit role in mathematics curricula, it was important to understand how this type of spatial thinking relates to mathematics. Examples of the digital training activities are provided in Figure 1, and the progression of the digital spatial training is provided in Table 2.

#### 2.2.3. The Embedded Intervention Group (Group C)

The Group C cohort participated in the embedded spatial visualization program. They engaged in the program for approximately 60 min per week for 14 weeks. The intervention replaced the measurement and geometry units that would usually be taught from the Australian Curriculum. The content of the program was delivered by the respective classroom teachers after undertaking professional learning.

**Figure 2:**
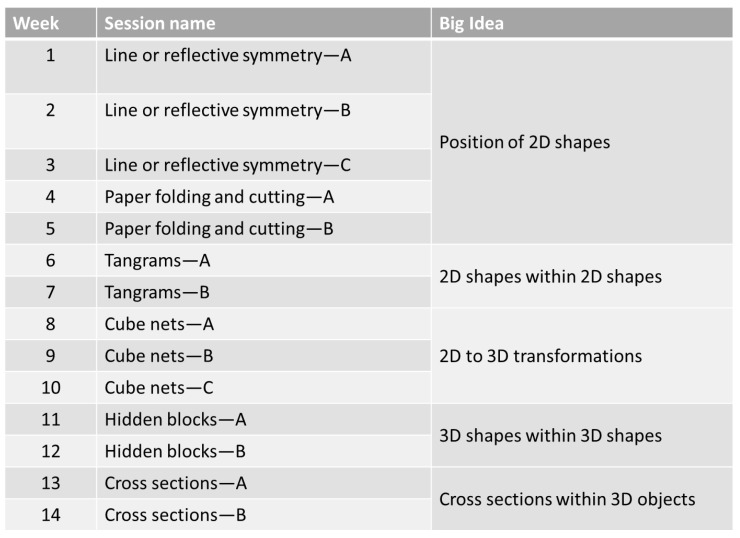
Overview of lesson structure for the embedded intervention.

During the intervention program, the participants were exposed to learning activities that encouraged spatial visualization, including open-ended tasks that could be solved with multiple solutions. Embedded intervention participants were introduced to learning experiences that evoked spatial reasoning through inquiry-based engagement—through both individual and cooperative-based experiences situated within the ELPSA pedagogical framework (see Appendix A for an example lesson within the ELPSA framework). Spatial visualization activities encouraged students to identify horizontal, vertical and incline reflections, determine the number of blocks within 3D objects and identify cross-sections of 3D objects. Additionally, students used mental folding to imagine how 2D nets could be folded and unfolded from 3D objects. Figure 2 presents an overview of the lesson content and structure for the embedded intervention. 

Across all lessons, students were encouraged to use visualization strategies to make predictions as part of their learning process rather than relying solely on concrete materials. The lesson included the use of the digital training platform that encouraged participants to practice mental manipulations of reflections and 3D mental folding. Digital spatial activities complemented the hands-on approaches, with the advantage of allowing participants to progress through activities at their own pace. 

### 2.3. Data Gathering Instruments and Procedures

#### 2.3.1. Spatial Reasoning

**Figure 3:**
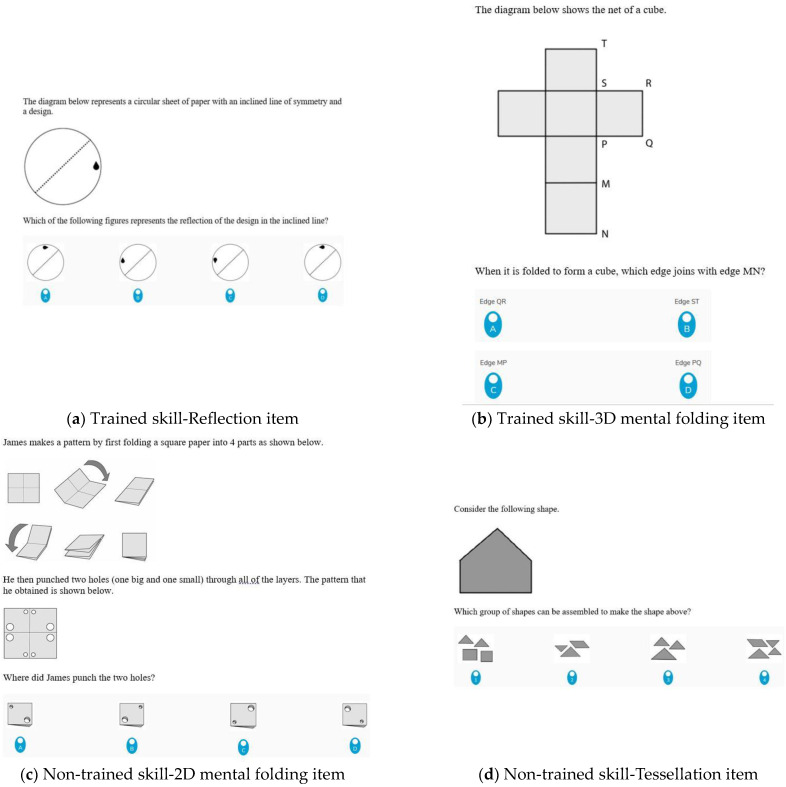
Examples of items within the two subsets of the Spatial Visualization instrument by spatial maneuver.

Items from the spatial visualization section of the 45-item Spatial Reasoning Instrument (SRI) were used to measure students’ spatial visualization (Ramful et al. 2017). The fifteen spatial visualization items were divided into two tests in order to measure spatial skills associated with the digital spatial training (8 items) and spatial skills not associated with the digital spatial training (7 items). The skills aligned to the digital spatial training component of the isolated intervention included reflection tasks that required students to reflect 2D shapes horizontally, vertically or on an incline (see Figure 3a). The second set of measures required students to construct 3D objects from 2D nets (3D mental folding) (see Figure 3b). The non-trained measure included items that required students to visualize the folding and unfolding of pieces of paper (2D mental folding) (see Figure 3c) and tessellation tasks that required students to determine how separate pieces can be put together to make a certain figure (see Figure 3d). The internal reliability produced a Cronbach alpha level of .65, which is deemed to be acceptable (Cronbach 1951). 

#### 2.3.2. Mathematics

A mathematics assessment was used to identify students’ mathematics performance before and after the intervention. It is a test developed from items released by Australia’s National Assessment Program (NAPLAN) Numeracy test with items drawn from released Year 3 and Year 5 NAPLAN tests across the years 2012–2016; consequently, the items were age appropriate. All of the items selected are analyzed by the Australian Curriculum, Assessment, and Reporting Authority (ACARA) for item reliability and content identification. 

The test contained 16 multiple-choice items associated with geometry and measurement (8 items), and number and algebra, statistics, and probability (8 items). Items require the application of mathematics knowledge instead of drill-and-practice procedures. Questions were given a score of 1 for correct or 0 for incorrect, with the highest potential score being 16. The internal reliability for this test had an alpha level of .70. 

#### 2.3.3. Test Administration

The spatial visualization and mathematics measures were delivered digitally to whole, intact classes by the classroom teacher via the digital platform developed for the study. The tests were untimed, but each one was completed by all students within 40 min. After a brief introduction, each child worked on the test individually. Testing was completed within the two weeks prior to the commencement of the intervention (pre-test) and within two weeks of its completion (post-test). 

#### 2.3.4. Data Analysis

Data were analyzed using SPSS 29 (IBM Corp. 2022). Descriptive statistics, correlations, and ANCOVAs were conducted to answer the first research question. Post hoc analysis was conducted with moderation analysis and mediation analysis using Model 4.2 of Hayes’ PROCESS SPSS macro (Model 4; Hayes 2015) to answer the second research question.

## 3. Results

Descriptive statistics for performance at pre- and post-test are shown in Table 3 (means and standard deviations) and Table 4 (Pearson Correlations). Following Cohen’s (1988) conventions, students’ math understanding was moderately correlated with both Reflection/3D Folding and 2D Folding/Tessellation spatial visualization measures at both pre-test and post-test. There was a strong correlation between the pre-test and post-test math measures (r = .700, *p* < .001). Despite the randomization of the sample, there were significant differences between the pre-test scores of the three groups for Reflection/3D Folding F(2,280) = 7.41, *p* < .001, η2 = 0.05; 2D Folding/Tessellation F(2,282) = 3.41. *p* = .03, η2 = 0.02; and math F(2,284) = 4.94. *p* = .008, η2 = 0.03. In each comparison, the isolated spatial intervention cohort had higher mean scores than the other two cohorts. Consequently, we used pre-test scores as a covariate in the following design to account for cohort variability and bias (Field 2009). 

Separate ANCOVAs were conducted to examine the effects of the intervention conditions on spatial visualization skills and (transfer to) math skills. With respect to the trained spatial skills (Reflection/3D Folding), there was a statistically significant difference in the mean scores of the three groups, F(2,276) = 10.05, *p* = .018, = η2 = 029. Post hoc analysis revealed that the differences were in favor of the embedded intervention (Group C) compared to BAU (Group A) and isolated intervention (Group B) (C > A; C > B; see Table 3). There was also a statistically significant difference in the mean scores of the three groups for the untrained spatial skills (2D Folding/Tessellation), F(2,278) = 3.38, *p* < 05, η2 = .023. In this analysis, there were differences in favor of both the embedded intervention (Group C) and the isolated intervention (Group B) compared to the BAU (Group A) (C > A; B > A, see Table 5). A third ANCOVA was conducted to determine if there were differences in the mean scores of the cohorts across the math measure F(2,283) = 3.90, *p* < .02, η2 = .027). Both the embedded and isolated intervention groups were statistically different to the BAU cohort (C > A; B > A, see Table 5), indicating math transfer for both the embedded and isolated training cohorts.

Despite explicit training on the Reflection/3D Folding visualization skills, the isolated intervention did not improve compared to the BAU cohort on this measure, yet they improved on the non-trained spatial skills of 2D Folding/Tessellation and on transfer to math. To understand this finding further, we considered the role of trained and non-trained spatial skills as mediators and the initial level of trained spatial skill as a moderator in the results of the isolated intervention (Group B). 

### 3.1. Trained Spatial Skills as a Mediator

Given the unexpected performance improvements of the isolated cohort, further analysis was undertaken to determine if there were any underlying mechanisms or processes that would help determine the transfer effects. To this point, it was worthwhile to understand whether the trained or untrained skills mediated mathematics performance. We anticipated that the trained skills would mediate performance in mathematics despite the students not making significant gains on these skills as a result of the intervention.

**Figure 4:**
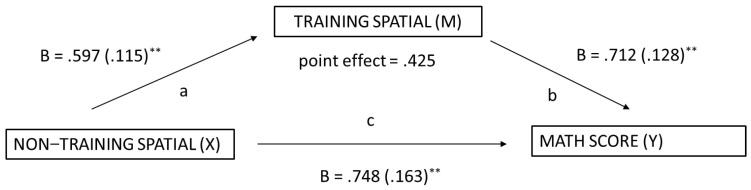
Spatial Training as a Mediator of Math Performance. ** *p* < .01.

The exploratory mediation analysis was undertaken using Model 4.2 of Hayes’ PROCESS macro (Model 4; Hayes 2015) for the isolated intervention (Group B) to determine whether the trained and untrained skills mediated mathematics performance. Significance was tested using a bootstrapping method with 5000 iterations. Indirect effects were calculated for each of the 5000 bootstrapped iterations, and the 95% confidence interval was determined by computing the indirect effect at the 2.5th and the 97.5th percentile. The first mediation analysis examined the relationship between non-trained spatial skills and math performance with trained spatial skills as the mediator variable. The total indirect effect was significant as the 95% confidence interval did not include zero (effect = .425, 95% CI [.225, .684]), suggesting that, taken together, the spatial training mediated the relation between non-trained spatial skills and math performance. Additionally, the Sobel mediation test was undertaken to confirm the indirect effect between non-training spatial skills and math performance via trained spatial skills. The test was statistically significant [Sobel test = 3.795, *p* < .001]. Figure 4 presents the regression Beta scores and Standard Errors for each association. 

**Figure 5:**
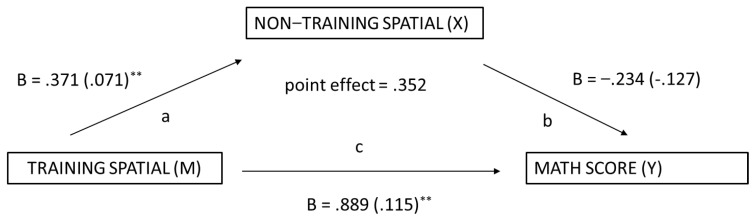
Non−Trained Spatial Skills as a Mediator of Math Performance. ** *p* < .01.

The second mediation analysis examined the relationship between trained spatial skills and math performance with non-trained spatial skills as the mediator variable (see Figure 5). The non-trained skills did not mediate performance in the math test [Sobel test = −0.921, *p* = .356].

### 3.2. Spatial Skill Level as a Moderator

Given that this cohort was exposed to specific spatial skills on a digital device, it was also beneficial to understand whether these transfer experiences were due to the student’s initial spatial thinking level. Elsewhere, for example, digital training was most effective for students with low spatial skills (Resnick and Lowrie, forthcoming). Consequently, to better understand the impact of the isolated intervention on math performance, a moderation analysis was conducted to determine the extent to which initial Reflection/3D Folding spatial skills (IV) and post-test math score (DV) were related. In line with Resnick and Lowrie (forthcoming), student performance at pre-test for the Reflection/3D Folding measure was separated into three spatial skill levels—low, mid, and high (see Table 6) for the moderator variable. These levels categorized students on their ability to successfully complete the mental spatial maneuvers required in the pre-test, with students scoring less than two correct, demonstrating a low ability to reflect images and fold 2D shapes into 3D objects.

For the isolated intervention (Group B), the overall model was statistically significant, R = .651, F(3,94) = 23.16, *p* < .001. The interaction effect between the independent variable (pre-test performance on Reflection/3D Folding spatial skill) and the moderator variable (skill level at pre-test) was statistically significant (β = .407, t(1,94) = 2.55, *p* = .012). The moderation on the dependent variable (post-test math performance) was statistically significant for students with mid- and high-level Reflection/3D Folding spatial skills. There was no moderation effect for students with low Reflection/3D Folding spatial skills (see Table 7).

## 4. Discussion

The current study is the first classroom-based, randomized-control trial to utilize both isolated spatial visualization skill training and embedded spatial visualization training in order to better understand the mechanisms that lead to math transfer. Recall that the isolated intervention trained students in skills that were needed to solve reflection and 3D mental folding tasks, while the embedded intervention developed other spatial skills in addition to those accessed through the digital training. Our central finding was that the two types of spatial training led to significant improvements in math outcomes when compared to the business-as-usual control. This finding is important because it indicates that both (i) laboratory-like (isolated) spatial training and (ii) embedded spatial training can be effective for transfer to math under typical whole-of-class conditions, an outcome not previously established (Hawes et al. 2022; Mix et al. 2021). Elsewhere, transfer for students of this age was more likely to occur when the intervention was established within a learning program, as opposed to a series of training tasks (Lowrie et al. 2018b). 

The design of the study allowed us to isolate specific spatial skills to better understand the mechanisms that lead to transfer. Such work is happening elsewhere (see Gilligan-Lee et al. 2020; Mix et al. 2021), but not under typical classroom conditions with the intervention administered by the classroom teacher. The students who were exposed to the complete embedded intervention had larger overall math effects for math transfer, in addition to improvements across both trained and non-trained spatial dimensions. The math transfer included both geometry and number-concept word-problems tasks. The pedagogical framework used in the integrated intervention (i.e., the ELPSA framework) presented learning experiences through a scaffolded assembly of learning activities to introduce and develop specific spatial visualization skills and practise these skills through digital training. The technological affordances also provided the students with immediate feedback loops, which Mix et al. (2021) noted was an important enhancement of their program success. The ELPSA framework embedded spatial visualization activities into the daily practices of teachers rather than relying only on the explicit teaching of spatial skills. Elsewhere, it has been argued that the transfer effects of classroom-based interventions need to be evaluated with respect to changes in teachers’ pedagogical practices as part of the program intervention (Lowrie et al. 2019). To this challenge, we can speculate that teachers can manage interventions that require the implementation of both digital training and concrete materials. 

In comparing the embedded intervention condition with the isolated intervention condition, we observed that the students in the isolated intervention cohort did not have statistically significant growth in the spatial skills trained in the digital platform when compared with the BAU cohort. Rather, improvements were observed in the visualization skills not explicitly trained. This finding suggests the isolated intervention had no close—yet some mid-transfer of spatial visualization skills—in addition to the far math transfer. It may be the case that the digital training equipped these students with better spatial working memory and/or improved general reasoning skills rather than the intended (specific) spatial skills scaffolded by digital engagement. These Reflection/3D Folding spatial skills did, however, have a significant indirect and mediated effect on the cohort’s math performance. By contrast, the non-trained spatial skills (2D Folding/Tessellation) in which this cohort did improve did not mediate beyond the effects of the skills presented in the digital training. Such findings support the tentative claim that the digital training supported math development through general reasoning development. This general reasoning development was likely effective when students formed and held mental representations (in the mind’s eye) for a longer duration, thus supporting the spatial visualization required to solve the non-trained items. Further, it may be the case that this more effective mental manipulation of images allowed students to cognitively access the more complex math tasks in ways that supported a more effective representation of the problem context. To this point, exposure to the digital experience could have made strategy use more purposeful for the relatively novel tasks (both the non-trained and math tasks). This contrasts with priming, where the unconscious activation of cognitive processing takes place because of an intervention and not as a result of conceptual change (Hawes et al. 2022). We propose that the relatively long-term duration of the study, and the fact that the post-test was conducted up to a week after the completion of the intervention, reduced the likelihood of a priming effect (see Hawes et al. 2022). We do note, however, that further research on the mechanisms for strategy transfer to mathematics needs to be undertaken in a more targeted form (as proposed by Cheng and Mix 2014) to better understand the impact of intentional strategy use and other unconscious processing of information. 

The current study also found that the level of initial spatial skill moderated the effect of the intervention for students in the isolated intervention cohort. Students with low, mid, and high initial spatial skills all improved in math; however, the digital training had the strongest effects on students with higher levels of initial spatial skills. Those students with low spatial skills likely found the games difficult to engage with and thus unsatisfying from a motivational perspective. Recall that this intervention cohort did not have a learning program to accommodate the digital training—relying on the feedback from the digital platform rather than a teacher to build and support the skill development. In other randomized classroom-based studies, the effects of the intervention tend to be moderated by spatial reasoning, with children with lower spatial reasoning making the most gains in numeracy (e.g., Resnick and Lowrie, forthcoming). This finding from the present study supports the claim that an integrated approach, which embeds spatial materials and digital tools within everyday learning, can support mathematics learning for students with varying levels of spatial skill development.

## 5. Implications and Directions for Future Research

It is challenging to substantiate the levels of fidelity and teacher agency in large classroom-based investigations. The situation and daily practices of the classroom, and classroom instruction, limit the controls that can be placed on a research design. Nevertheless, most of our intervention studies are based in intact classrooms with the programs administered by the student’s own classroom teacher—given it is the best way to know if the intervention works. For the current study, our design approach provided the opportunity to determine whether an integrated intervention would be more effective than an intervention which relied on a scaffolded training program of specific spatial skills. Although the integrated intervention provided strong gains in students’ spatial reasoning and transfer to math, these gains were not substantially different to an intervention that provided the training support only (especially for far math transfer). In part, this may be because the classroom teachers’ usual pedagogical practices and mathematics instruction complemented the game training; after all, the game training intervention was posited within each teacher’s daily practice.

Future research might examine the relative contributions of different aspects of the spatial learning programs used in the current study to develop more targeted and efficient programs. For example, such designs might consider a third intervention, which involves the administration of the learning program only. Given the fact that the current intervention bundled several spatial visualization skills, it is beyond the scope of the study to characterize specific causal mechanisms. Nevertheless, we speculate about our key outcome measures. The spatial skills required to develop students’ understanding of symmetry (i.e., vertical, horizontal and incline symmetry of 2D shapes) and net construction (i.e., interpreting 2D shapes and 3D objects) seem to support far transfer to mathematics. In an integrated program, this transfer occurs via substantial improvement in these and other spatial visualization skills, namely, skills involving paper folding and tessellating 2D shapes. For the game training cohort, the symmetry and net construction skills mediated improvements in students’ math performance. Consequently, this study goes some way to helping the field understand the spatial mechanisms that support transfer to math.

## Figures and Tables

**Table 1:** Intervention design of the study.

Group	Group Title	Intervention	N
A	Business-as-usual	Control: Followed the curriculum of the state. Completed approximately 4 h of mathematics per week for 14 weeks.	97
B	Isolated Intervention	Digital Spatial Skills: Followed the curriculum of the state. Completed approximately 3.5 h of mathematics and 40 min (+/−10 min) of digital spatial skills per week for 14 weeks.	98
C	Embedded Intervention	Spatial Visualization Program and Digital Spatial Skills: Followed the curriculum of the state. Completed approximately 3 h of mathematics and 60 min (+/−10 min) of spatial visualization and digital spatial skills per week for 14 weeks.	92

**Table 2:** Digital spatial training progression for reflection and 3D mental folding skills.

Reflections	3D Mental Folding
Week 1. Students choose the image which has been reflected over horizontal and vertical lines. The images are colour; the line is displayed. There are 3 answer options.	Week 8. Students choose the configuration of shapes that will fold to make a net. Three options are provided. There are 11 questions for the 11 nets.
Week 2. Students choose the image which has been reflected over a horizontal and vertical line. The images are colour; the line is not displayed. There are 4 answer options.	Week 9. As above, but four options are provided, and nets are presented in random orientation.
Week 3. Students choose the image which has been reflected over an inclined line. The images are colour; the line is displayed. There are 3 answer options.	Week 10. Students choose the opposite face to the star on the given net. These nets are all 1:4:1 nets.
Week 4. Students choose the image which has been reflected over a horizontal, vertical, and inclined line. The images are colour; the line is displayed. There are 4 answer options.	Week 11. As above, but these nets are combinations of 1:3:2, 2:3:1, 2:2:2, and 3:3 nets.
Week 5. Students choose the image which has been reflected over a horizontal, vertical, and inclined line. The images are colour; the line is not displayed. There are 4 answer options.	Week 12. As above, but these are combinations of all net configurations.
Week 6. Students choose the image which has been reflected over a horizontal, vertical, and inclined line. The images are black and white; the line is displayed. There are 3 answer options.	Week 13. Students are shown a net configuration with a symbol on one of its faces. They must rotate a given 3D cube to see it has two symbols on it. One of the symbols corresponds to the symbol on the net. They must position the other symbol on the correct face of the net. These are all 1:4:1 nets. It begins with opposite faces and then moves to adjacent faces.
Week 7. Students choose the image which has been reflected over a horizontal, vertical, and inclined line. The images are black and white; the line is not displayed. There are 4 answer options.	Week 14. As above, but these are a combination of 1:3:2, 2:3:1, and 2:2:2 nets. The last two questions have no fixed symbol on the net, so the students must position both symbols to correspond with the cube.

**Table 3:** Mean (standard deviation) of student performance on outcome measures.

Outcome Variable		Pre-Test			Post-Test		
		Business-as-Usual (BAU) Control	Isolated Spatial Intervention	Embedded Spatial Intervention	Business-as-Usual (BAU) Control	Isolated Spatial Intervention	Embedded Spatial Intervention
		97	98	92	97	98	92
Spatial Visualization:							
	Reflection/3D Folding	3.24 (1.59)	4.04 (1.79)	3.23 (1.66)	3.96 (1.87)	4.48 (1.97)	4.53 (1.92)
	2D Folding/Tessellation	2.43 (1.22)	2.88 (1.33)	2.45 (1.51)	2.58 (1.44)	3.23 (1.53)	2.95 (1.45)
Transfer skill: Math		9.81 (2.55)	10.11 (2.65)	8.94 (2.71)	10.19 (2.49)	11.07 (2.83)	10.25 (2.70)

**Table 4:** Correlations for the spatial visualization and math measures by test.

	Outcome Measure	1	2	3	4	5
Pre-test	1. Reflection/3D Folding	--				
	2. 2D Folding/Tessellation	.338 **	--			
	3. Math	.428 **	.461 **	--		
Post-test	4. Reflection/3D Folding	.563 **	.334 **	.490 **	--	
	5. 2D Folding/Tessellation	.406 **	.438 **	.513 **	.452 **	--
	6. Math	.430 **	.422 **	.700 **	.579 **	.514 **

** *p* < .01.

**Table 5:** Post hoc analysis for ANCOVAs by intervention cohort.

(I) Group	(J) Group	Mean Difference (I-J)	Std. Error	*p* Value
Trained spatial skills				
Group A BAU	Group B Isolated	.005	.233	.984
	Group C Embedded	−.574 *	.233	.014
Group B Isolated	Group C Embedded	−.578 *	.234	.014
Non-trained spatial skills				
Group A BAU	Group B Isolated	−.449 *	.196	.023
	Group C Embedded	−.391 *	.197	.048
Group B Isolated	Group C Embedded	.058	.198	.769
Math transfer				
Group A BAU	Group B Isolated	−.664 *	.274	.016
	Group C Embedded	−.672 *	.281	.017
Group B Isolated	Group C Embedded	−.008	.282	.976

* *p* < .05

**Table 6:** Reflection/3D Folding skill levels at pre-test.

Level of Spatial Skill	N	%	Score Range/8
Low	23	23.5	<2
Mid	36	36.7	2–4
High	39	39.8	>4
Total	98	100.0	

**Table 7:** Moderation analysis: Trained spatial skill level moderates the effects of the isolated intervention transfer to math.

Spatial Level	Effect	SE	t	p	LLCI	ULCI
Low	.402	.222	1.81	.071	−.038	.843
Medium	.809	.137	5.89	.001	.536	1.08
High	1.21	.198	6.13	.001	.823	1.61

## Data Availability

Data are protected due to student privacy; for access to the data please contact the corresponding author.

## Appendix A

**Table A1:** Spatial Visualization lesson structured under the ELPSA Framework—Lines of symmetry lesson (Lowrie et al. 2018b).

ELPSA Framework	Student Work Samples	Student Voice
**Experience:** What is symmetry? Students begin with more familiar reflections of letters and symbols along the *y* and *x* axes. They are then asked to consider reflections of similar letters and symbols along the diagonal axis.		“Symmetry is something like butterfly wings, they look the same on both sides and it might be a shape that is a mirror shape that is rotated”
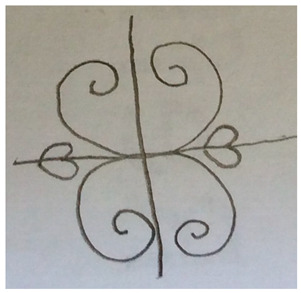
**Language:** What are the language conventions associated with symmetry? Line of symmetry; reflections; mirror image; visualize; fold; *y* and *x* axes; diagonal axis; vertical; horizontal; imagine→predict→experiment→check;		“How to flip it on the y or x axis so I was trying to visualize the mirror” “Today we revised on our spatial awareness. I understand now what the *y* and *x* axes are. The *y* axis is when it goes these ways and the *x* axis when it goes these ways ”
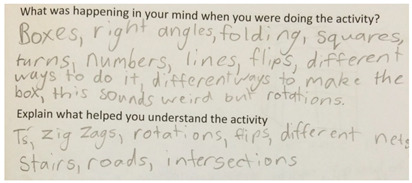
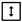
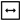
**Pictorial:** Students represented their ideas about lines of symmetry in a drawing. They are required to draw a square with letters in the bottom left corner and a vertical (*y* axis) line of symmetry. Teacher example below. Move onto horizontal (*x* axis) line of symmetry and then diagonal line of symmetry.		“I was imagining a mirror reflecting the picture” “I was imagining in my mind the paper folding and where it would print” “I worked it out by picturing it in my mind and trying to think of the page was folded diagonally and thinking what the “T” was doing during that time” “I was visualizing if I flipped it, what shape would it make and where would it be?” “When I was doing this activity I was flipping the images in my mind to get the reflections correct. I was also imagining a mirror on the line so I could draw the reflections correctly.”
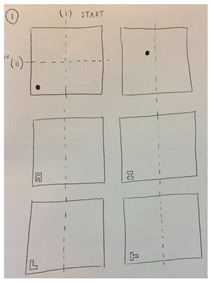
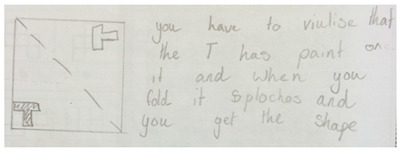
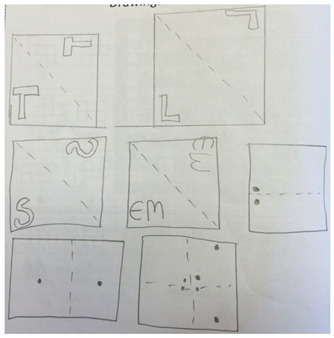
**Symbolic:** Symbolic stage requires analytic thinking. Here, students need to recognize conventions associated with lines of reflection on vertical, horizontal, and diagonal axes. Students begin to reason that for reflections on the *x* and *y* axes, horizontal stays horizontal and vertical stays vertical. However, with diagonal reflections, horizontal moves to vertical and vertical moves to horizontal. See images below for the concept of perpendicularity.		“I did have to think a bit harder to remember that if the curve in the umbrella on the left is facing left, then the one on the right will be opposite.” “I was imagining a mirror on the fold of the page. Using visual measurements to make it as accurate as possible” “When I was doing the activity I remembered that we talked about the arrow and how it went from ↑ to ←.” “I used my hand a first then I realised I could just reflect then rotate which was much easier.” (*diagonal reflection*)
